# Supplementary figures and images for: The small acid-soluble proteins of Clostridioides difficile regulate sporulation in a SpoIVB2-dependent manner
Source: PLoS Pathog. 2024 Aug 30;20(8):e1012507. doi: 10.1371/journal.ppat.1012507 (PMC11392383; doi:10.1371/journal.ppat.1012507)

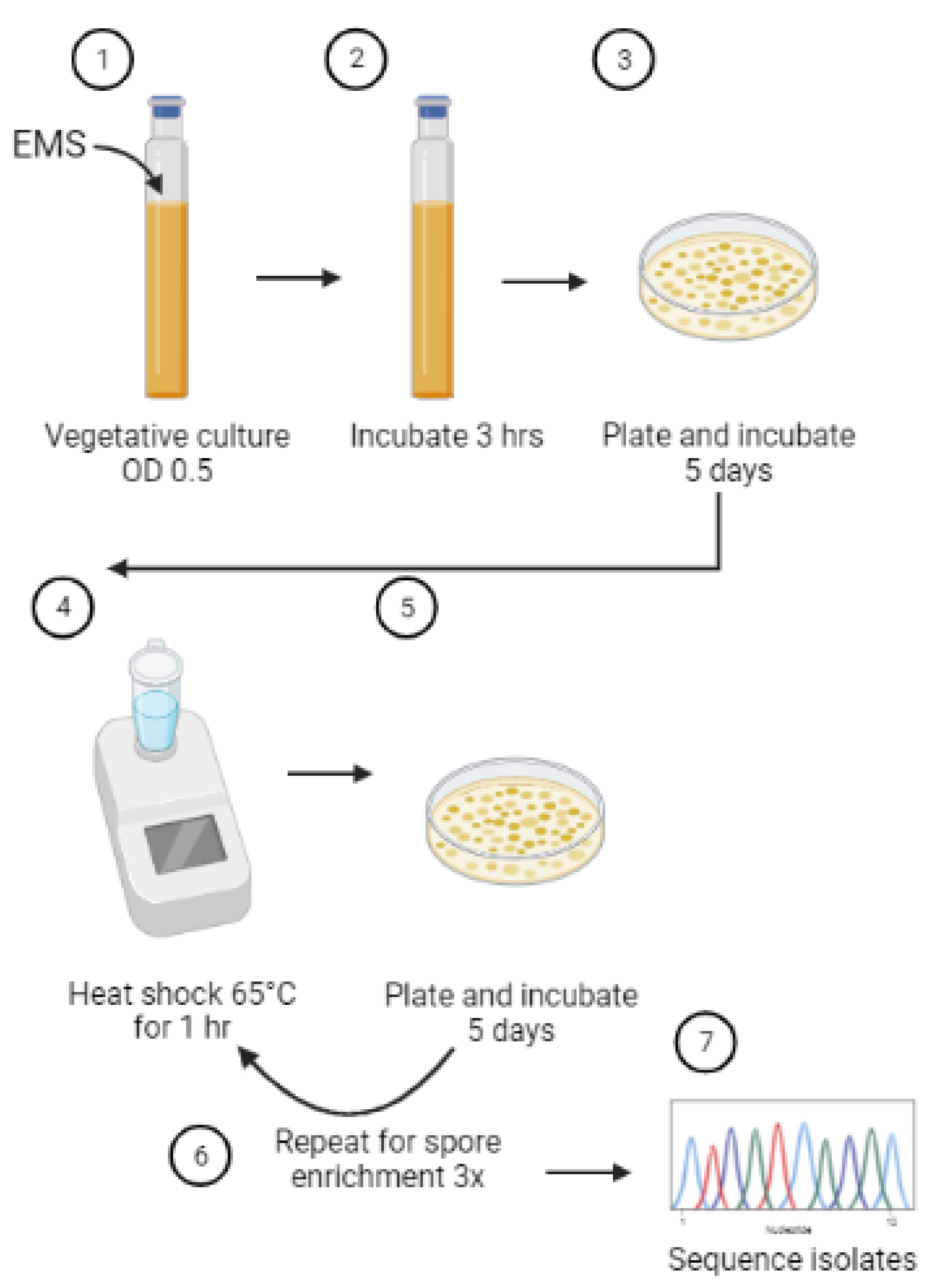

Supplement: S1 Fig — EMS was added to logarithmically growing cultures and incubated for 3 hours. The resulting cells were spread on sporulation medium (70:30) and incubated for 5 days. Subsequently, the growth was harvested, purified, and then then heat shocked at 65°C for 1 hour. After heat treatment, the samples were plated onto 70:30 and again incubated for 5 days. This enrichment process was repeated 3 times before individual colonies were isolated, phenotypes confirmed, and DNA sent for whole genome re-sequencing. The figure was created with BioRender.com. (TIF) [file ppat.1012507.s001.tif]

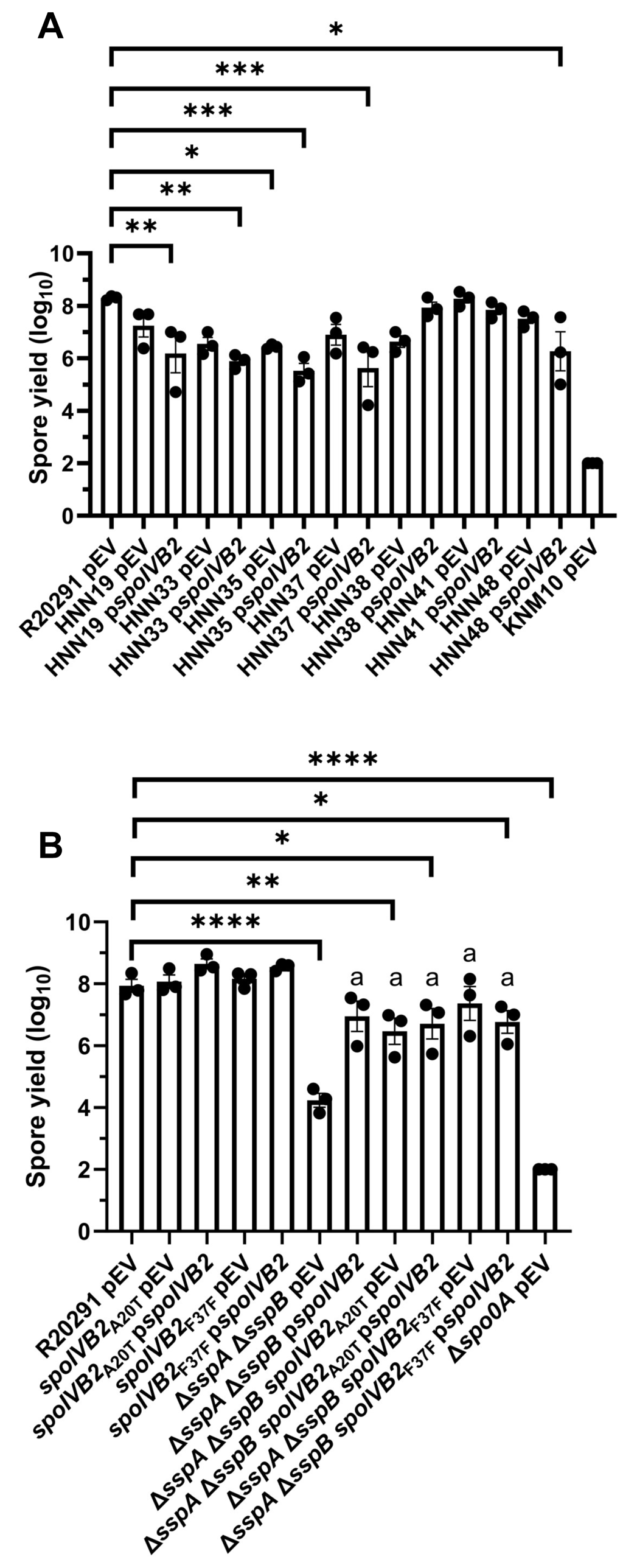

Supplement: S2 Fig — Spore yield of the indicated strain was determined as described in Fig 1. pEV indicates an empty vector. A) Strains isolated during EMS. B) Clean strains containing generated spoIVB2 alleles. All data represents the average of three independent experiments. Statistical analysis by one way ANOVA with Šίdák’s multiple comparison test. * P<0.05, ** P<0.01, *** P<0.001, **** P<0.0001. B) a P<0.0001 in comparison to C. difficile ΔsspA ΔsspB. (TIF) [file ppat.1012507.s002.tif]

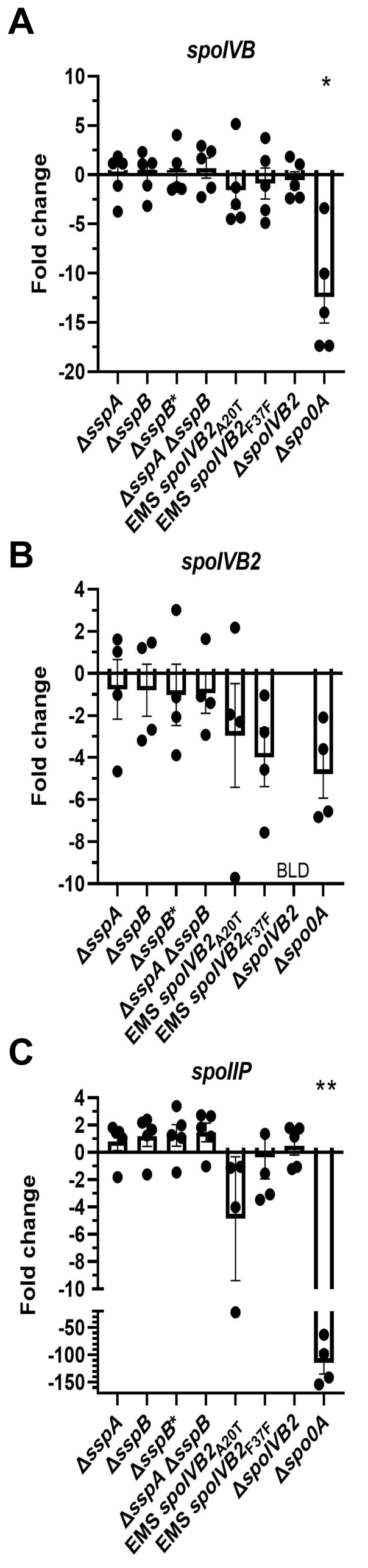

Supplement: S4 Fig — Strains were grown on sporulation medium for 11 hours before RNA extraction. qPCR was performed using SYBR green. Transcripts for the following genes were determined: A) spoIVB, B) spoIVB2, C) spoIIP. Fold change from R20291 was determined with the ΔΔCT method using rpoA transcripts as the internal control. All data represents the average of five independent experiments. Statistical analysis by one way ANOVA with Dunnett’s multiple comparison test with the mutant strains compared to wild type. * P<0.05, ** P<0.01, *** P<0.001, **** P<0.0001. (TIF) [file ppat.1012507.s004.tif]
