# Supplementary material for: The small acid-soluble proteins of Clostridioides difficile regulate sporulation in a SpoIVB2-dependent manner
Source: PLoS Pathog. 2024 Aug 30;20(8):e1012507. doi: 10.1371/journal.ppat.1012507 (PMC11392383; doi:10.1371/journal.ppat.1012507)
Supplement: S1 Table — (DOCX) [file ppat.1012507.s006.docx]

**Supplement Table 1. Primers used in this study.**

| Primer Name | Sequence |
| --- | --- |
| 5'sspA_MTL | ttatcaggaaacagctatgaccgcggccgcttagatgaggaaaaactggataa |
| 3'sspA_up | ttatttataactatctgttgctttttccaggttgattaccttccttctgttta |
| 5'sspA_down | aataaattaaacagaaggaaggtaatcaacctggaaaaagcaacagatagt |
| 3' sspA_xylR | tgcaggcttcttatttttatgctagctcgagctattgaacttggaaatgagag |
| CRISPR_sspA_165 | gtgtgctataattaaactgtaaaacgcgtgactaaaaaattagttgaaagttttagagctagaaatagcaagttaaaataaggctagtccgttatcaacttgaaaaagtggcaccgagtcggtgctttttttctatggagaaatctagatcagcatgatgtctgactagacgcgtaagctctgcaactatttttagat |
| 5'traJ | gcgaggaagcggaagagcgcccaatacgcagggccccctgcttcggggtca |
| 3'traJ | aatttatctacaatttttttatcctgcagggggcccgatcggtcttgccttg |
| CRISPR_sspA_135 | taattaaactgtaaaggtaccagagaaaatggttatgtagggttttagagctagaaatagcaagttaaaataaggctagtccgttatcaacttgaaaaagtggcaccgagtcggtgctttttttctatggagaaatctagatcagcatgatgtctgactagacgcgtaagctctgcaactat |
| 5' sspB UP | atttttttatcaggaaacagctatgaccgcggccgcttttaaaatatcatccatattat |
| 3' sspB UP | tgtcaaaatttactatttattttccagccacctcaaataattagtttatgatg |
| 5' sspB DN | tgttagacatcataaactaattatttgaggtggctggaaaataaatagta |
| 3' sspB_xylR | atgcaggcttcttatttttatgctagctcgagatacttgtctattttttcagtaa |
| CRISPR_sspB_144 | aattaaactgtaaaggtaccagagaaaatggatatgttgggttttagagctagaaatagcaagttaaaataaggctagtccgttatcaacttgaaaaagtggcaccgagtcggtgctttttttctatggagaaatctagatcagcatgatgtctgactagacgcgtaagctctgcaacta |
| 5' CDR20291_0714 UP | ttatcaggaaacagctatgaccgcggccgcccttgatgccctgggtctatac |
| 3' CDR20291_0714 UP | cttatttatttatattatttacaacatccgttgcataaaacacctctttct |
| 5' CDR20291_0714 DN | ttaataagaaagaggtgttttatgcaacggatgttgtaaataatataaataa |
| 3' CDR20291_0714 DN | gcaggcttcttatttttatgctagctcgagggaacagcattaggaagtcc |
| CDR20291_0714 gRNA 3 | gcattcaaggagggggtaccgtatctattttattaaataggttttagagctagaaatagc |
| 3' gRNA_change | ccatctaaaaatagttgcagagcttacgcgtctagtcagacatcatgctgatctag |
| 5' tn916.traJ | tctgcagattacctaataatttatctacattccctttagcctgcttcggggtcattat |
| 5'Tn916ori_gibson | cggaagagcgcccaatacgcagggccctaacatcttctatttttcccaaatc |
| 3' tn916.traJ | cgaaaaaatcgctataatgaccccgaagcaggctaaagggaatgtagataaattattag |
| 5' CDR20291_0714 | ttttttatcaggaaacagctatgaccgcggccgctgctatctccttttccttg |
| 3' CDR20291_0714 | gtgccaagcttgcatgtctgcaggcctcgagttacaacatccatttaataaatac |
| 3' 0714_S301A | ttattatcttgaactattggagtacctgccataccttgtacaataccacc |
| 5' 0714_S301A | ctcaaactggtggtattgtacaaggtatggcaggtactccaatagttcaag |
| 3' sspA.pJS116 | tgccaagcttgcatgtctgcaggcctcgagctatctgttgctttttccag |
| 3' sspAsspB | ggaactgataatatggatgatattttaaaactatctgttgctttttccagccatttg |
| 5' sspAsspB | caaatggctggaaaaagcaacagatagttttaaaatatcatccatattat |
| 3'sspBpJS116 | cagtgccaagcttgcatgtctgcaggcctcgagttattttccagccatttgtc |
| 5' rpoA | taaaggtagaggttatgtttctgct |
| 3' rpoA | tttgaccaactcttgtgttttcc |
| 5' sspA_qPCR | caaaagaggctttaaaccaaatgaa |
| 3' sspA_qPCR | attttctcttgcagtaaggtttcctt |
| 5' sspB_qPCR | aacagaacagtagttccagaagcaaa |
| 3' sspB_qPCR | caacatatccattttctctagctgttaag |
| 5'sleC_qPCR | ttgaagcaagacaaggagttccc |
| 3'sleC_qPCR | cgaaaccagtaggaggaggtaatgg |
| 5' spoVT_qPCR | agagaaggagaccctttagagat |
| 3' spoVT_qPCR | ctgttatcaacactccatatcctagt |
| 5' pdaA_qPCR | tggtaaacagccatcacctataa |
| 3' pdaA_qPCR | tccactttcatatccagcatca |
| 5'spoIVA_qPCR | ggatagaacaagagatgagataccc |
| 3'spoIVA_qPCR | ctgctgccttttcaaatgtc |
| 5' spoIVB_qPCR_1 | ttcagagctaggtataagtggtaat |
| 3' spoIVB_qPCR_1 | tgcggtcttccaacttctatt |
| 5' spoIVB2_qPCR_1 | agctcaaactggtggtattgt |
| 3' spoIVB2_qPCR_1 | catgtgacactgctccgatta |
| 5' spoIIP_qPCR_1 | catactcatggatgtgagacttattcaa |
| 3' spoIIP_qPCR_1 | accccatcctttgctatctaaagc |
| 5' dpaA_qPCR | actgttattgggggagacctgc |
| 3' dpaA_qPCR | ggaacttttggatagtgcttcagc |
| 5' spoVAC_qPCR | agctggagctggttctataattcc |
| 3' spoVAC_qPCR | catagccttctcttttatactccattgc |
| 5' spoVAD_qPCR | tgacagctcagtggacagttacag |
| 3' spoVAD_qPCR | tttggaccgtctccattagga |
| 5' spoVAE_qPCR | gtttaatagcccaagtaatgatggatt |
| 3' spoVAE_qPCR | acaccagttgttacatacgttaccataa |
| 3' luciferase_ssrA_pHN149 | aagcttgcatgtctgcaggcctcgagtcatcatgcagcaagtgcataattttcatcattagctgctagaatttcttcaaaaagtctat |
| 3' luciferase_pHN149 | gccaagcttgcatgtctgcaggcctcgagtcatcatagaatttcttcaaaaag |
| 3' PsspA_BS49 | gttgttgctgttacctgagttattgttagccatgttgattaccttccttctgt |
| 5' sspA_BS49 | acacaaaataaattaaacagaaggaaggtaatcaacatggctaacaataactcagg |
| 3' sspA_BS49 | ccagtgccaagcttgcatgtctgcaggcctcgagttagaattgtcctccgcc |
| 3' spoIVB2 F36F | attttgtgcataaattaaattatttgagaagaaatataataaaaataaaaatgttaaaac |
| 5' spoIVB2 F36F | tacaattgttttaacatttttatttttattatatttcttctcaaataatttaatttatgc |
| 3' spoIVB2 F37.UUA | aattttgtgcataaattaaattatttgataaaaaatataataaaaataaaaatgttaaa |
| 5' spoIVB2 F37.UUA | caattgttttaacatttttatttttattatattttttatcaaataatttaatttatgcac |
| 3' spoIVB2 F37.UUG | aattttgtgcataaattaaattatttgacaaaaaatataataaaaataaaaatgttaaa |
| 5' spoIVB2 F37.UUG | caattgttttaacatttttatttttattatattttttgtcaaataatttaatttatgcac |
| 5’sacB_UP | ttatcaggaaacagctatgaccgcggccgcgtcgactagttctttaggcccg |
| sacB_3’_XhoI | aagcttgcatgtctgcaggcctcgagttatttgttaactgttaattgtccttgttcaagg |
| 3’ PsspA_spoIVB2 | ttaaagtattatttttaaaatgaaaatttttaagttgcatgttgattaccttccttctg |
| 5’ spoIVB2_PsspA | acaaaataaattaaacagaaggaaggtaatcaacatgcaacttaaaaattttcatt |
| 5’ spoIVB.pHN149 | acaatttttttatcaggaaacagctatgaccgcggccgctttattgtcttcgaatatac |
| 3’ PspoIVB_spoIVB2 | aagtattatttttaaaatgaaaatttttaagttgcatatatccatctactcctatgc |
| 5’ spoIVB2_PspoIVB | aatacataataatacagcataggagtagatggatatatgcaacttaaaaattttcat |
| 5’ PspoIVB2_pHN149 | atttttttatcaggaaacagctatgaccgcggccgctatttatttttatgaaaactaagg |
| 3’ PspoIVB(100)_PspoIVB2 | cttaataagtgatttttaatacataatatgaaaacacctctttcttatta |
| 5’ PspoIVB2_PspoIVB | ctattattaaaataatttaataagaaagaggtgttttcatattatgtattaaaaatcact |
| 3’ PsspA_PspoIVB2 | ttaattcaggtaatttttagcaattaaaacctgaaaacacctctttcttatta |
| 5’ PsspA_PspoIVB2 | ttattaaaataatttaataagaaagaggtgttttcaggttttaattgctaaaaa |
| 3’ spoIVB2_homol | tatttttaaaatgaaaatttttaagttgcataaaacacctctttcttattaaattat |
| 5’ spoIVB2_gene_homol | ttaaaataatttaataagaaagaggtgttttatgcaacttaaaaattttcattttaa |
| 3’ spoIVB2end_lrgBit | tcccaatcacccacaaaatcttcaagtgtaaacaccaacatccatttaataaatacacc |
| 5’ lrgBit_spoIVB2end | ctgtaggttatggtgtatttattaaatggatgttggtgtttacacttgaagattttgtgg |
| 3’ spoIVB2_bitLuc | ccaatcacccacaaaatcttcaagtgtaaacaccataaaacacctctttcttattaaatt |
| 5’ bitLuc_PspoIVB2 | attattaaaataatttaataagaaagaggtgttttatggtgtttacacttgaagattttg |
| 5’ spoIVB2_theo | ttttttatcaggaaacagctatgaccgcggccgcgatttgtcttcatttttatcttta |
| 3’ spoIVB2_theo | gccagtgccaagcttgcatgtctgcaggcctcgagaaatatcaaagttattaatttgac |

CRISPR targeting sequence is in **bold.**
