# Supplementary material for: The small acid-soluble proteins of Clostridioides difficile regulate sporulation in a SpoIVB2-dependent manner
Source: PLoS Pathog. 2024 Aug 30;20(8):e1012507. doi: 10.1371/journal.ppat.1012507 (PMC11392383; doi:10.1371/journal.ppat.1012507)
Supplement: S2 Table — (DOCX) [file ppat.1012507.s007.docx]

**Supplement Table 2. Strains and plasmids used in this study.**

| **Strain** | **Description** | **Reference** |
| --- | --- | --- |
| *E. coli* DH5a | Cloning strain | [1] |
| *E. coli* HB101 pRK24 | Conjugal donor strain, Amp^R^ | [2] |
| *E. coli* MB3436 | *recA^+^ E. coli* strain | Gift from Dr. Michael Benedik |
| *B. subtilis* BS49 | *Tn*916 donor strain, Tet^R^ | [3] |
| *C. difficile* R20291 | Wild type, ribotype 027 | [4] |
| *C. difficile* CD630∆*erm* | Wild type, ribotype 012 | Gift from Dr. Daniel Paredes-Sabja (Texas A&M University) |
| *C. difficile* KNM10 | R20291 *spo0A* CRISPR-Cas9 mutant | [5] |
| *C. difficile* HNN03 | R20291 *sspA* CRISPR-Cas9 mutant | [6] |
| *C. difficile* HNN04 | R20291 *sspB* CRISPR-Cas9 mutant with an *sspA*_G52V_ allele (called *sspB** throughout this manuscript) | [6] |
| *C. difficile* HNN05 | R20291 *sspA* and *sspB* CRISPR-Cas9 double mutant | [6] |
| *C. difficile* HNN17 | R20291 *sspB* CRISPR-Cas9 mutant | [6] |
| *C. difficile* HNN19 | EMS isolate from treatment of HNN04 | This study |
| *C. difficile* HNN22 | EMS isolate from treatment of HNN04 | This study |
| *C. difficile* HNN26 | EMS isolate from treatment of HNN04 | This study |
| *C. difficile* HNN28 | EMS isolate from treatment of HNN04 | This study |
| *C. difficile* HNN32 | EMS isolate from treatment of HNN05 | This study |
| *C. difficile* HNN33 | EMS isolate from treatment of HNN05 | This study |
| *C. difficile* HNN35 | EMS isolate from treatment of HNN05 | This study |
| *C. difficile* HNN37 | EMS isolate from treatment of HNN05 | This study |
| *C. difficile* HNN38 | EMS isolate from treatment of HNN05 | This study |
| *C. difficile* HNN39 | EMS isolate from treatment of HNN05 | This study |
| *C. difficile* HNN40 | EMS isolate from treatment of HNN05 | This study |
| *C. difficile* HNN41 | EMS isolate from treatment of HNN05 | This study |
| *C. difficile* HNN43 | CD630Δ*erm sspB* CRISPR-Cas9 mutant | This study |
| *C. difficile* HNN45 | CD630Δ*erm sspA* CRISPR-Cas9 mutant | This study |
| *C. difficile* HNN46 | CD630Δ*erm sspA* and *sspB* CRISPR-Cas9 double mutant | This study |
| *C. difficile* HNN48 | EMS isolate from treatment of HNN05 | This study |
| *C. difficile* HNN49 | R20291_*0714* (*spoIVB2*) CRISPR-Cas9 mutant | This study |
| *C. difficile* HNN51 | EMS isolate from treatment of HNN05 | This study |
| *C. difficile* HNN57 | R20291 *spoIVB2*_F37F_ | This study |
| *C. difficile* HNN60 | R20291 *spoIVB2*_A20T_ | This study |
| *C. difficile* HNN64 | R20291 Δ*sspA* Δ*sspB spoIVB2_A20T_* | This study |
| *C. difficile* HNN73 | R20291 Δ*sspA* Δ*sspB spoIVB2*_F37F_ | This study |
|  |  |  |
| **Plasmid** | **Description** | **Reference** |
| pMTL84151 | *E. coli* – *C. difficile* shuttle vector | [7] |
| pMTLYN4 | *traJ* containing plasmid | [8] |
| pJS116 | *B. subtilis* – *C. difficile* shuttle vector | [9] |
| pKM197 | CRISPR plasmid with *xylR* promoter driving *cas9* | [10] |
| pMB81 | BitLuc containing plasmid | [12] |
| pJB09 | *cas*9 containing plasmid for the 2-plasmid CRISPR system | [13] |
| pJB14 | Targeting plasmid for the 2-plasmid CRISPR system | [13] |
| pJB94 | Theophylline allelic exchange base plasmid | [14] |
| pJB96 | pHN149 with *sacB* between *Not*I and *Xho*I cut sites, for easy selection of inserts | This study |
| pHN14 | R20291 *sspB* promoter region and gene | [6] |
| pHN30 | R20291 *sspA* and *sspB* complement | [6] |
| pHN120 | CD630Δ*erm sspA* targeted CRISPR vector, gRNA 1398 | This study |
| pHN121 | CD630Δ*erm sspB* targeted CRISPR vector, gRNA 1186 | This study |
| pHN122 | CDR20291_0714 promoter region and F37F allele | This study |
| pHN123 | CDR20291_0714 promoter region and A20T allele | This study |
| pHN127 | CDR20291_0714 promoter region and WT allele | This study |
| pHN131 | CD630Δ*erm sspA* targeted CRISPR vector with *TraJ oriT,* gRNA 165 | This study |
| pHN132 | CD630Δ*erm sspB* targeted CRISPR vector with *TraJ oriT*, gRNA 144 | This study |
| pHN138 | CD630Δ*erm sspA* targeted CRISPR vector with *TraJ oriT*, gRNA 135 | This study |
| pHN145 | CDR20291_0714 promoter region and S301A allele | This study |
| pHN146 | CDR20291_0714 promoter region and F37F, S301A allele | This study |
| pHN147 | CDR20291_0714 promoter region and A20T, S301A allele | This study |
| pHN149 | pMTL84151 based plasmid that also contains the *Tn916 oriT* (base plasmid that can be conjugated through *E. coli* or *B. subtilis* conjugal donors) | This study |
| pHN152 | CD630Δ*erm sspA* promoter region and gene | This study |
| pHN153 | CD630Δ*erm sspA* and *sspB* promoter region and gene | This study |
| pHN157 | CDR20291_0714 targeted CRISPR vector, gRNA 3 | This study |
| pHN176 | CD630Δ*erm sspB* promoter region and gene | This study |
| pHN208 | CDR20291_0714 promoter region and F36F | This study |
| pHN218 | CDR20291_0714 promoter region and F37L (UUA codon) | This study |
| pHN219 | CDR20291_0714 promoter region and F37L (UUG codon) | This study |
| pHN220 | *sspA* promoter region and *sspA* gene from *B. subtilis* BS49 | This study |
| pHN271 | *spoIVB2*A20T theophylline allelic exchange | This study |
| pHN272 | *spoIVB2*F37F theophylline allelic exchange | This study |
| pHN312 | *sspA* promoter driving *spoIVB2* expression | This study |
| pHN329 | *spoIVB* promoter driving *spoIVB2* expression | This study |
| pHN330 | *spoIVB2* and *spoIVB* promoters driving *spoIVB2* expression | This study |
| pHN331 | *spoIVB2* and *sspA* promoters driving *spoIVB2* expression | This study |
| pHN335 | *spoIVB2* promoter with *spoIVB2* attached to *bitLuc* (luciferase) and tagged with *ssrA* | This study |
| pHN336 | *spoIVB2* promoter with *spoIVB2*_A20T_ attached to *bitLuc* (luciferase) and tagged with *ssrA* | This study |
| pHN337 | *spoIVB2* promoter with *spoIVB2*_F37F_ attached to *bitLuc* (luciferase) and tagged with *ssrA* | This study |
| pHN338 | *spoIVB2* promoter with *bitLuc* (luciferase) and tagged with *ssrA* | This study |
| pHN339 | *spoIVB2* promoter with *bitLuc* (luciferase) | This study |

1. Hanahan D. Studies on transformation of *Escherichia coli* with plasmids. J Mol Biol. 1983;166(4):557-80. Epub 1983/06/05. doi: 10.1016/s0022-2836(83)80284-8. PubMed PMID: 6345791.

2. Ma NJ, Moonan DW, Isaacs FJ. Precise manipulation of bacterial chromosomes by conjugative assembly genome engineering. Nature protocols. 2014;9(10):2285-300. doi: 10.1038/nprot.2014.081.

3. Bouillaut L, McBride SM, Sorg JA. Genetic Manipulation of *Clostridium difficile*. Current Protocols in Microbiology2011.

4. Stabler RA, He M, Dawson L, Martin M, Valiente E, Corton C, et al. Comparative genome and phenotypic analysis of *Clostridium difficile* 027 strains provides insight into the evolution of a hypervirulent bacterium. Genome Biol. 2009;10(9):R102. Epub 2009/09/29. doi: 10.1186/gb-2009-10-9-r102. PubMed PMID: 19781061; PubMed Central PMCID: PMCPMC2768977.

5. McAllister KN, Martinez Aguirre A, Sorg JA. The selenophosphate synthetase, *selD*, is important for *Clostridioides difficile* physiology. BioRxIV. 2021. doi: 10.1101/2021.01.06.425661.

6. Nerber HN, Sorg JA. The small acid-soluble proteins of *Clostridioides difficile* are important for UV resistance and serve as a check point for sporulation. PLoS Pathog. 2021;17(9):e1009516. Epub 2021/09/09. doi: 10.1371/journal.ppat.1009516. PubMed PMID: 34496003.

7. Heap JT, Pennington OJ, Cartman ST, Minton NP. A modular system for *Clostridium* shuttle plasmids. J Microbiol Methods. 2009;78(1):79-85. Epub 20090513. doi: 10.1016/j.mimet.2009.05.004. PubMed PMID: 19445976.

8. Ng YK, Ehsaan M, Philip S, Collery MM, Janoir C, Collignon A, et al. Expanding the repertoire of gene tools for precise manipulation of the *Clostridium difficile* genome: allelic exchange using *pyrE* alleles. PLoS One. 2013;8(2):e56051. Epub 2013/02/14. doi: 10.1371/journal.pone.0056051. PubMed PMID: 23405251; PubMed Central PMCID: PMCPMC3566075.

9. Sorg JA, Sonenshein AL. Bile salts and glycine as cogerminants for *Clostridium difficile* spores. J Bacteriol. 2008;190(7):2505-12. Epub 2008/02/05. doi: 10.1128/JB.01765-07. PubMed PMID: 18245298; PubMed Central PMCID: PMCPMC2293200.

10. Bhattacharjee D, Sorg JA. Factors and Conditions That Impact Electroporation of *Clostridioides difficile* Strains. mSphere. 2020;5(2). Epub 2020/03/07. doi: 10.1128/mSphere.00941-19. PubMed PMID: 32132157; PubMed Central PMCID: PMCPMC7056809.

12. Baloh MaS, J.A. . *Clostridioides difficile* SpoVAD and SpoVAE Interact and Are Required for Dipicolinic Acid Uptake into Spores. J Bacteriol. 2021;203(21). doi: <https://doi.org/10> .1128/JB.00394-21.

13. Brehm JN, Sorg JA. Plasmid Sequence and Availability for an Improved *Clostridioides difficile* CRISPR-Cas9 Mutagenesis System. Microbiol Resour Announc. 2022;11(12):e0083322. Epub 20221107. doi: 10.1128/mra.00833-22. PubMed PMID: 36342279; PubMed Central PMCID: PMCPMC9753633.

14. Brehm JN, Sorg JA. Plasmid sequence and availability for an improved *Clostridioides difficile* CRISPR-Cas9 mutagenesis system Microbiol Resour Announc. 2022;11(12 ). doi: 10.1128/mra.00833-22.
