# Supplementary material for: The small acid-soluble proteins of Clostridioides difficile regulate sporulation in a SpoIVB2-dependent manner
Source: PLoS Pathog. 2024 Aug 30;20(8):e1012507. doi: 10.1371/journal.ppat.1012507 (PMC11392383; doi:10.1371/journal.ppat.1012507)
Supplement: Attachment — Submitted filename: 2024_PLOS_Reviewer_Comments.docx [file ppat.1012507.s010.docx]

**We thank the reviewers for their helpful comments. We have made substantial changes to this manuscript based on their suggestions. Importantly, we have made the luciferase fusion to the full length *spoIVB2* and the F37F and A20T alleles and these hybrids are driven by the native promoter. This suggestion has resulted in a new hypothesis that now cleans up prior data in the paper where we showed that expression of wildtype *spoIVB2* resulted in some suppression. We hypothesize that the SASPs are activating gene expression (*spoIVB2*) so that some SpoIVB2 protein accumulates in σ^G^ timing. The F37F and A20T alleles result in a much greater abundance of protein (as read by luminescence). This new data is backed up by more new data where we make different promoter fusions to *spoIVB2* and show that adding some σ^G^ expression can suppress the defect in sporulation that is observed for the Δ*sspA*/*sspB* double mutant strain. These helpful suggestions have opened new avenues for pursuit and we are grateful to the reviewers for such suggestions.**

Reviewer #1: Previous work in this lab found that small acid soluble spore proteins (SASP) not only play a major role in spore UV resistance, but are also important for normal sporulation. In the current work they now find that in the absence of the two major SASP, SspA/B: i) the spore cortex layer is not formed; ii) a number of mutations can suppress the sporulation defect, in particular a number of mutations in the spoIVB2 protease, deletion of which also has a sporulation defect leading to no cortex synthesis; iii) several of the spoIVB2 mutants suppressed the sporulation defect due to the absence of SspA/B synthesis in a way that was not dependent on the likely proteolytic activity of SpoIVB2 that is essential for normal sporulation, but the SpoIVB2 mutants did not alter sporulation of wild-type spores.

The observations noted above are certainly interesting, and further analyses are carried out in attempts to determine: a) the cause(s) of the effects of the suppressor mutations  and b) why the absence of most SASP leads to asporogeny, with effects on likely relevant genes' transcription  +/- SspA/B, as well some possible translational effects, although the rationale for using only small pieces of spoIVB2 for this the translational study were not made clear.. However, no new definitive answers to the latter questions were obtained.

..

Reviewer #2: The manuscript by Nerber et al. provides an interesting follow-up study to the authors’ previous report detailing that the major small acid soluble proteins (SASPs) of Clostridioides difficile play critical roles during spore formation beyond their role in protecting DNA from UV damage. Loss of both SspA and SspB in C. difficile arrests sporulation at a previously undefined stage of sporulation (but likely after engulfment), suggesting that the production of the SASP proteins constitutes a checkpoint during C. difficile spore formation to ensure that functional spores are produced. In the current manuscript, the authors demonstrate that their ∆sspA∆sspB (or ∆sspB*) mutants appear to be defective in cortex formation, explaining their significant defect in producing functional spores. Using a suppressor screen, the authors identify mutant alleles in spoIVB2 that suppress the sporulation defect of the SASP mutants in C. difficile. Remarkably, several of the

suppressors they identified lead to synonymous mutations in spoIVB2 that may reduce the translational efficiency of the spoIVB2 transcript. C. difficile spoIVB2 encodes a sporulation-induced protease with homology SpoIVB in Bacillus subtilis, which regulates a proteolytic signaling cascade in B. subtilis that does not apply in C. difficile and plays a second poorly defined role in potentially producing the germ cell wall of B. subtilis spores. The authors show that C. difficile SpoIVB2 is critical for spore formation and that loss of the protein or its catalytic activity appears to result in a defect in cortex synthesis. Thus, their data suggest that SpoIVB2 plays a dual role in regulating sporulation in C. difficile (analogous to how B. subtilis SpoIVB plays two discrete roles during spore formation, albeit by different mechanisms).

Based on their analyses, the authors propose a model in which the DNA binding activity of SASPs functions to repress spoIVB2 transcription at later stages of sporulation, acting as a negative feedback mechanism to down-regulate the production of SpoIVB2. Accordingly, loss of the major SASPs leads to the aberrant production of SpoIVB2 (likely over-production) due to the inability to repress spoIVB2 transcription; this subsequently impairs spore formation by blocking cortex synthesis. Aberrant production of SpoIVB2 at later stages of spore formation leads to a defect in cortex synthesis and thus constitutes a checkpoint on spore formation.

The authors present compelling genetic data demonstrating that the spoIVB2 A20T and F37F alleles can suppress the ∆sspA∆sspB and ∆sspB* mutants by expressing the spoIVB2 mutant alleles on a plasmid in the presence of a WT spoIVB2 allele in the native locus (merodiploid) and recapitulating the phenotypes of the suppressors that they isolated in their genetic screen. In Figure 8 they show that different synonymous mutations in the Phe37 codon differentially affect the suppression, providing further evidence that codon 37 (and 36) impact the ∆sspA∆sspB mutants.

--------------------

<b>Part II – Major Issues: Key Experiments Required for Acceptance

Reviewer #1: Interpreting the data in some figures is more difficult than it should be - for example in Fig. 2A only sporulation % for the complemented strains is shown, and not the effects of the sspA, deltasspB* and sspA/sspB mutations that are being complemented, although two of the mutants were analyzed in Fig. 1B; similar comment about Fig. 4A.

**We have repeated the assays as suggested and included the control strains as requested.**

In Fig. 4A, expression of wt spoIVB2 from a plasmid suppressed the spo defect (I think) of an sspA/B double deletion. How does this fit with the idea that in the sspA/B mutant there is excess SpoIVB2 protein made?

**The expression of WT *spoIVB2* partially suppressed the sporulation defect in the *sspA/sspB* mutant. Based on new data in this manuscript, our original hypothesis that the SASPs are repressing expression of *spoIVB2* was incorrect. New evidence points toward the SASPs activating *spoIVB2.***

The results from the PCR analyses of gene expression +/- SASP in Fig. S3 were somewhat disappointing, and with the suppressor mutants might be confounded by the presence of multiple EMS caused mutations. Why not make the appropriate spoIVB2 mutant in a clean background for testing? Might RNA seq be more useful in examining transcription of genes at the best possible time in sporulation of strains +/- SspA/B, perhaps using analysis of a fluorescent fusion in a gene coexpressed with sspA/B to find the optimal times to look!? Indeed this analysis might find some general evidence of many genes who's transcription is repressed by SASP.

**Overall, the results are confounded by our inability to sync sporulation. Because *C. difficile* sporulates heterogeneously and asynchronously, the RNA from the extracted cells represents all stages of sporulation, not just the stage we are interested in. While other mutations within the EMS-generated suppressor strains may contribute to the variability, the main issue is that the sample is heterogeneous.**

**Unfortunately, the same problem arises for RNA-seq; this is a goal, however better tools are needed to hyper-sporulate or synchronize sporulation to have a clear picture of transcripts levels at each stage of sporulation. Even in the studies that determined the program of sporulation (Fimlaid & Shen 2013 and Saujet & Martin-Verstraete 2013), the authors required mutations in defined regulators of sporulation to help fight through the noise of the heterogeneous population.**

Reviewer #2: Despite this intriguing data, a few pieces of data do not support their model. (1) They were unable to show that the synonymous mutations decrease the translational efficiency of spoIVB2 (using a luciferase reporter construct), which they propose as a reason for why the mutant alleles suppress the SASP mutants. (2) They did not detect statistically significant transcriptional changes in the expression of spoIVB2 in the ∆sspA∆sspB or ∆sspB* parental strains as proposed by their model. (3) Most importantly, if the spoIVB2 suppressor alleles they isolated decrease the aberrant production of SpoIVB2 in the SASP mutant background, one would not expect that the suppressor alleles should work in a merodiploid stain, where the WT allele would be over-expressed according to their model. (In addition, they observed differential suppression in their two different  SASP mutant strains, which is hard to interpret).

A few experiments might provide more support for their model

(1) Given that the forespore has a different metabolic context than vegetative cells, expressing their reporter constructs from the native forespore-specific promoter might allow them to detect differences.

**Thank you for this suggestion. The indicated experiment was done and showed that our original hypothesis was wrong. With the constructs expressed from the native promoter, we found a marked increase in *spoIVB2* F37F and A20T but not the wildtype allele. This indicates that the F37F and A20T alleles are increasing the abundance of SpoIVB2 during sporulation. Based upon this data and the data using different sporulation-specific promoters, we now hypothesize that the SASPs are activating sporulation. Thank you very much for this suggestion.**

Alternatively, demonstrating that SpoIVB2 protein levels increase in the SASP mutant background and decrease in the suppressor strains would have provided direct evidence for their model, although these experiments are probably more technically challenging if there is a subtle difference.

**We have attempted to extract and blot for SpoIVB2 from cultures. However, SpoIVB2 is only present in a sporulating strain (i.e. it is not found in vegetative cells or in dormant spores) and, with the heterogeneous population, it was not practically possible to extract enough protein to be useful in this assay. i.e. the amount of extract needed to load on the gel to see SpoIVB2 resulted in blown out bands from the amount of total protein present in the sample. With this, it was not possible to compare between strains.**

(2) Using RNA-Seq might allow them to more systematically test their hypothesis, but this is not essential.

**As stated above for Reviewer #1, we are unable to perform this experiment until better tools become available / are developed to synchronize sporulation in *C. difficile*.**

Alternatively, putting spoIVB2 under the control of a SigG-regulated promoter so that it is expressed likely inappropriately later during sporulation might lead to a defect in spore formation similar to a ∆sspA∆sspB mutant.

**Thank you for this suggestion. We have performed this experiment. Sporulation is restored when *spoIVB2* is expressed under the *spoIVB* promoter (a σ^G^-dependent promoter with lower expression levels) but not the *sspA* promoter (a σ^G^-dependent with high expression levels) in a *spoIVB2* mutant. Towards the reviewers point, when expressed in R20291, only the construct expressing *spoIVB2* during σ^F^ and σ^G^ (sspA) reduced sporulation. With the new hypothesis that SASPs are activating *spoIVB2,* it seems that the data points to the continued expression during σ^G^ not affecting the cells, but when highly expressed (*e.g.*, the *sspA* promoter) sporulation is affected.**

(3) Since the authors used EMS mutagenesis to isolate the suppressors, and there were many other mutations in a given strain, moving the suppressor alleles into a clean ∆sspA∆sspB or ∆sspB* mutant background would provide more rigorous support for the importance of the mutant alleles.

**We generated the A20T and F37F *spoIVB2* alleles in the wild type and the Δ*sspA/sspB* mutant background. The sporulation assays showed that these alleles could rescue the Δ*sspA/sspB* double mutant sporulation phenotype.**

Related to this point, how did the authors decide which mutations to exclude based on their sequencing analyses? Why did they focus exclusively on spoIVB2?

**We explored this specific mutation because *spoIVB2* had a hit in 7/15 independent trials and the CDR20291­­_0714 (*spoIVB2*) was already involved in sporulation based on its annotation. We are still exploring the other mutations (*e.g.*, the RNAP mutations, but this work is outside the scope of this paper).**

Unless the authors can provide more experimental evidence for their proposed model, a revised manuscript should consider a possible alternative model that could explain the differences they observe, namely that the mutant alleles alter the expression of a small non-coding RNA found within the spoIVB2 gene that regulates spore formation. This could explain why they did not observe transcriptional changes in the spoIVB2 transcript in the SASP mutants but why the synonymous mutations and A20T allele can suppress the phenotype (and why they may see a difference expressing the mutant alleles in the context of a WT spoIVB2 background.)

**The small non-coding RNA hypothesis is unlikely because catalytic activity is necessary for restoration of sporulation so the mechanism is likely related to this function. Also, we now hypothesize that the A20T and F37F alleles are rescuing translationally rather than transcriptionally.**

An important caveat to the interpretation of their interesting study is that the parental SASP mutant strains are not provided in many of their functional assays measuring spore formation, so it is difficult to interpret their complementation analyses. While other investigators combine the sporulation efficiencies measured for a given strain across multiple replicates (using experiments where not necessarily all strains were measured in the same assay, so some strains are measured more than three independent times), a complication to this approach is the change in media composition that markedly impacted their sporulation efficiencies. Due to this limitation, unfortunately, it seems like repeating the sporulation efficiency assays with the parental controls may be necessary.

**The experiments were repeated with the suggested strains.**

In terms of the missing controls in the figures,

Fig. 2A – the ΔsspAΔsspB and ΔsspB* controls are missing in this figure, so it is difficult to see how well the B. subtilis sspA complements the sspA defect in R20291.

**The experiment was repeated with these strains.**

Fig. 2B – the ΔsspA control is missing

**Experiments were re-done with the Δ*sspA* strain.**

In Fig. 4A – it is hard for the reader to know if the expression of WT spoIVB2 impacts the sporulation frequency in the ΔsspB* or ΔsspA ΔsspB mutant background, since these parental mutants are not presented in this panel for comparison.

**The experiment was re-done with these strains.**

Fig. 8. Controls are missing from each panel.

**The experiment was re-done with these strains.**

**--------------------**

<b>Part III – Minor Issues: Editorial and Data Presentation Modifications</b></br>Please use this section for editorial suggestions as well as relatively minor modifications of existing data that would enhance clarity.<br/><br/>

Reviewer #1: There were a number of little "niggling" things about the manuscript as follows.

1) There were no page or line numbers.

**Edited as suggested.**

2) pg 6, l 7 fb "whose germinated spores".

**Edited as suggested.**

3) pg 7, l 8 fb - ", in the double deletion".

**Edited as suggested.**

4) pg 9, l 7 but the coat also looks anomalous too as would probably be expected.

**Edited as suggested.**

5) pg 9, l 4 fb - while this says 14 suppressor strains, there are 18 in Table 1.

**Thank you for pointing this out. The number of suppressor strains and the various mutations were recounted and there are 15 instead of the 14.**

6) pg 10, l 9 - If there are two spoIVBs in C. difficile, do they both look to do the same thing? Is the spoIVB mutation asporogenous? Is a double mutant even more asporogenous?

**Since we originally submitted this work, Martins, *et. al.* Mol Micro 2024 adresses these questions and we have now cited it in this manuscript.**

7) pg 11, l 7/8 fb - But not clear how the PspoIVb2 plasmid will "restore" the spo defect in the sspA/B deletion, as it is already "spo".

**These alleles were identified in suppressor screen for the sporulation phenotype, therefore it should restore spore formation, as in it will now sporulate where it could not before.**

8) pg 13, l 1 - don't understand this sentence.

**This line is referencing data from our previously published paper. We previously found that there was very little DPA in the purified “spores” from these strains. It is likely that the DPA was able to “escape” because the cortex layer was not in place to prevent leakage of core contents.**

9) references - in some bacterial species names are not in italics.

**Edited as suggested.**

10) reference 17 needs P. Eichenbergers's name.

**Edited as suggested.**

11) Supplement pg 1, l 6 fb - Fold change relative to what? Reference for the method needed.

**More details on how the samples were compared were added to the figure legends. The reference for this method is found in the method section when explaining how the qPCR was performed and analyzed.**

2Reviewer #2: Aside from these comments, there are some minor changes in wording that should be added to correct some descriptions of the literature in an otherwise clearly written manuscript. (Adding line numbers and page numbers would help in the review process.)

Introduction: 2nd page – a sigG mutant in the 630 background can complete engulfment (Pereira et al. PLoS Genet 2013). A sigK mutant can localize the basement layer of the coat – so please add “visible” in front of “coat layer”. Notably, engulfment is NOT a SigG-regulated process – it is dependent on SigF and SigE (Serrano et al., Ribis et al. Mol Micrb 2018).

**A σ^G^ mutant in strain 630 does not fully engulf as it does not complete membrane fission (it does not exclude Hoechst dye) (Fimlaid 2013). The wording was changed so it now suggests that engulfment is driven by σ^G^.**

**Visible was added in front of coat layer.**

- it would be helpful to indicate that the mutations result in loss of the protein (e.g. “loss of SigF results in a strain…”)

**Edited as suggested.**

- final sentence of the intro – change “repress” to “represses”

**Edited as suggested.**

Results:

First paragraph – the term “sporulation frequency” should be changed to “sporulation efficiency,” which should be defined as the ratio of ethanol-resistant spores in the mutant relative to WT. It is likely that all the strains induce sporulation at similar levels (frequency), so efficiency reflects the difference in functional spores production between strains.

**The data was changed to spore yield - the total number of spores produced between the strains - so this description was changed accordingly.**

Section on isolating suppressor mutants:

- Consider moving the description of the catalytic mutant phenotypes to after describing the impact of the WT spoIVB2 alleles (would improve the flow of the paragraph and make the lack of a phenotype with over-expression of spoIVB2 more apparent). Consider starting the next paragraph, with “In contrast, when the spoIVB2 alleles were…”

**Edited as suggested.**

Section “Isolating suppressor mutations of the SASP mutant phenotypes,” Paragraph 1:

“After removing cellular debris, the cultures were plated on a medium supplemented with germinant and allowed to sporulate” –the authors likely mean “allowed to germinate”

**Edited as suggested.**

“Testing the impact of the suppressor alleles on spoIVB2 expression” – spoIVB2 should be the gene and not the protein (i.e. not capitalized).

**Edited as suggested.**

The paragraph that follows should be written in the past tense. Suggest that the text be modified to: “Though sspA and sspB levels were largely unchanged, there was a slight increase in transcript levels for spoIVA, which encodes a protein involved in spore coat localization and proper cortex synthesis.” (Please add the appropriate references)

**Edited as suggested.**

-              The wording in the first paragraph is also a little vague, making it unclear which mutants were tested until cross-referencing the text with the supplemental figures. The authors could clarify which mutants were examined in the text to streamline the reader’s ability to interpret the data.

**Edited as suggested.**

Last results section: please change the tense to past tense so that it reads “Because the translational differences between spoIVB2 alleles were minimal…”

**Edited as suggested.**

Discussion

Third paragraph: Cortex is under the control of SpoVD (Srikhanta et al. Nat Micrb 2020, Alabdali et al. 2021. and likely SpoVE based on B. subtilis. Cortex is modified by PdaA, GerS, and CwlD.

**Edited as suggested- replacing synthesized with modified.**

Fourth paragraph: SigE and SigF are made in both compartments (forespore and mother cell) because their respective genes are activated by Spo0A.

**Edited as suggested.**

- The summary of B. subtilis SpoIVB function could be summarized in a couple of sentences (the amount of detail is probably not needed to help with the flow of the Discussion)

**Some details were removed as suggested.**

However, the Discussion should mention that SpoIVB in B. subtilis is secreted into the intermembrane space between the mother cell and forespore, but it is unclear whether this occurs in the authors’ model.

**Edited to add secretion.**

Regarding the figures, the labeling of the graphs makes it ambiguous what the parental strain is for the strains that are carrying plasmids. The labeling for Figure 8 is much clearer and should be applied to other figures to improve readability.

**Edited as suggested**

- it would be nicer to have more concentrated images of the sporulating cells, as there are only a few visible in a given field (not necessary to change, but it would be nice)

**This was left unchanged because it was the field of view that was most concentrated while showing the incomplete spore phenotype.**
